# Design, Synthesis and Characterization of a Highly Effective Inhibitor for *A*nalog-*S*ensitive (*as*) Kinases

**DOI:** 10.1371/journal.pone.0020789

**Published:** 2011-06-17

**Authors:** Michael Klein, Montse Morillas, Alexandre Vendrell, Lars Brive, Marinella Gebbia, Iain M. Wallace, Guri Giaever, Corey Nislow, Francesc Posas, Morten Grøtli

**Affiliations:** 1 Medicinal Chemistry, Department of Chemistry, University of Gothenburg, Göteborg, Sweden; 2 Cell Signalling Unit, Department de Ciències Experimentals i de la Salut, Universitat Pompeu Fabra (UPF), Parc de Recerca Biomèdica de Barcelona (PRBB), Barcelona, Spain; 3 Department of Cell and Molecular Biology, University of Gothenburg, Göteborg, Sweden; 4 Department of Pharmaceutical Sciences, University of Toronto, Toronto, Canada; 5 Department of Molecular Genetics, University of Toronto, Toronto, Canada; Stanford University, United States of America

## Abstract

Highly selective, cell-permeable and fast-acting inhibitors of individual kinases are sought-after as tools for studying the cellular function of kinases in real time. A combination of small molecule synthesis and protein mutagenesis, identified a highly potent inhibitor (1-Isopropyl-3-(phenylethynyl)-1*H*-pyrazolo[3,4-*d*]pyrimidin-4-amine) of a rationally engineered Hog1 serine/threonine kinase (Hog1^T100G^). This inhibitor has been successfully used to study various aspects of Hog1 signaling, including a transient cell cycle arrest and gene expression changes mediated by Hog1 in response to stress. This study also underscores that the general applicability of this approach depends, in part, on the selectivity of the designed the inhibitor with respect to activity versus the engineered and wild type kinases. To explore this specificity in detail, we used a validated chemogenetic assay to assess the effect of this inhibitor on all gene products in yeast in parallel. The results from this screen emphasize the need for caution and for case-by-case assessment when using the Analog-Sensitive Kinase Allele technology to assess the physiological roles of kinases.

## Introduction

Protein kinases have a crucial role in most, if not all, signaling pathways and regulate diverse cellular functions, such as cell-cycle progression, apoptosis, metabolism, differentiation, cell morphology and migration, and secretion of cellular proteins [1]. Our present understanding of the majority of cellular signal transduction takes the form of wiring diagrams in which many of the component parts have been identified, and to some extent the relative position of the components in a given pathway, but beyond this static snapshot view, little is known about the details of their dynamic operation. A critical piece of this puzzle is an understanding of how external and internal inputs are sensed in a time-dependent manner to effect a given signaling output. Highly selective, cell-permeable and fast-acting inhibitors of individual kinases would allow for the systematic investigation of the *in vivo* cellular function of a kinase in real time. Protein kinases share common sequences and structural homology in their ATP-binding site. The fact that many kinases share a highly conserved catalytic domain complicate the search for ATP competitive kinase inhibitors with sufficient specificity [2]. However, this conserved domain can be leveraged to deliver high selectivity by orthogonal targeting [3]. This approach involves modifying a kinase inhibitor to disrupt its binding affinity for its native target and subsequent mutation of a protein to allow it to recognize the orthogonal inhibitor. Shokat and colleagues have extensively used this “analog-sensitive” approach to study a range of protein kinases [4]. Recently, this chemical genetic approach has been used to identify four novel physiological substrates of Hog1 kinase [5], to show that the catalytic activity of Hog1 prevents cross talk between the high-osmolarity glycerol (HOG) pathway and both the pheromone response and invasive growth pathways [6], as well as to define the signaling properties underlying the HOG pathway [7]. We wanted to explore orthogonal targeting in order to develop selective and fast acting kinase inhibitors that would allow us to study the dynamic behavior of kinases in the HOG pathway. Herein we report the design, synthesis and evaluation of an orthogonal inhibitor that is able to inhibit *as* kinases efficiently and can be used to study signal transduction events that occur within minutes, e.g. gene expression and cell cycle studies.

The HOG pathway of the yeast *Saccharomyces cerevisiae* is a MAPK signaling pathway and is the functional homolog of the stress activated MAPK JNK and MAPK p38 pathways of mammals [8]. Because there is a high degree of conservation of these cascades, the yeast HOG pathway is a good model to study osmotic adaptation processes. The HOG pathway consists of two upstream osmosensing branches, the Sln1 and Sho1 branches, and a downstream MAP kinase cascade including the Ssk2/22, Ste11 MAP3K, the Pbs2 MAPKK and Hog1 MAPK [9]. Activation of the Hog1 MAPK elicits an extensive program required for cell adaptation which includes profound changes in gene expression. Specifically, Hog1 regulates gene expression by activation of specific transcription factors but also through chromatin binding, Hog1 recruits chromatin modifying/remodeling activities to stress-responsive genes altering their expression [8], [10]. In addition, environmental stressors (e.g. changes in osmolarity) critically affect progression through the cell cycle [9], [11], [12].

To develop an analog-sensitive inhibitor of an engineered Hog1 kinase, we selected the pyrazolopyrimidines as they represent an excellent scaffold for targeting the protein kinase family due to their structural similarity to the adenine moiety of ATP, furthermore, the scaffold has been shown to have activity against multiple kinase subfamilies. For example, different chemical substitutions around this scaffold result in increased selectivity in the inhibition of KDR [13], Src [14], and EGF [15] kinase families. Furthermore, this scaffold has previously been used to make orthogonal inhibitors [16]. We present here the design and synthesis of a novel orthogonal inhibitor based on the pyrazolopyrimidine that effectively inhibits a Hog1as kinase, and is able to dissect the transient cell cycle arrest and regulation of gene expression mediated by Hog1 in response to stress.

## Results and Discussion

**Figure 1:**
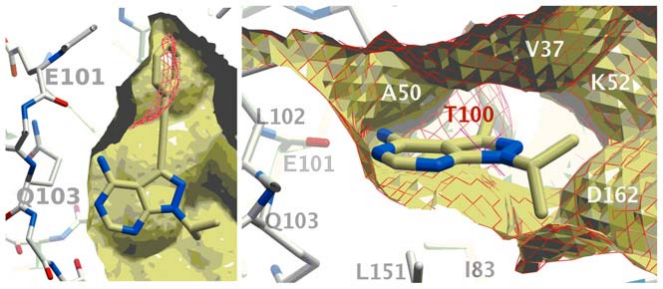
Inhibitor docked to Hog1. Compound **6a** docked to the homology models of wild-type (red mesh) and T100G mutant (ball-and-stick and yellow surface) Hog1, shown as cross sections from two angles, rotated 90 degrees around the horizontal axis. Compound **6a** was designed to occupy the region available only for the T100G mutant (red mesh near phenyl ring) for specificity.

Because of its central role in cellular homeostasis and the implication of human homologs in diverse disease states, we selected Hog1 as the target of our mutant kinase-inhibitor pair design. Sequence alignment analyses identified the conserved T100 as a gatekeeper residue in Hog1 [5], [6]. Visual inspection of the binding pocket of an initial homology model of Hog1, using the structure of human p38 in the absence of a ligand (pdb code 1p38) for a template, indicated that a narrow path leads to a buried cavity within the ATP binding domain (Figure 1).

The cavity size and shape is comparable to that of a phenyl group, and mutation of T100 for a glycine would widen the pocket further (Figure 1). We therefore sought a compound that was based on the pyrazolopyrimidine structure, having a phenyl ring attached to it via a spacer of the appropriate length. Candidate compounds were manually docked into the binding site and the geometries were optimized in torsion space using an all-atom representation of both ligand and receptor, keeping the receptor fixed. 1-NM-PP1, a commercially available ATP competitive *as*-inhibitor was compatible with our model, but did not fit as well as other compounds into the ATP binding site of Hog1as. The resulting model complex that best matched our specifications included a two-carbon, triple-bonded linker (compounds with the general structure **6**, Figure 1). The triple bound would place the benzene ring in such orientation that it fills up the lipophilic pocket that becomes accessible upon mutation. At the same time, the heterocyclic moiety can make similar interactions with the hinge area as would ATP. In the wild-type kinase the non-mutated gatekeeper residue should block access to the lipophilic pocket (indicated in red).

**Figure 2:**
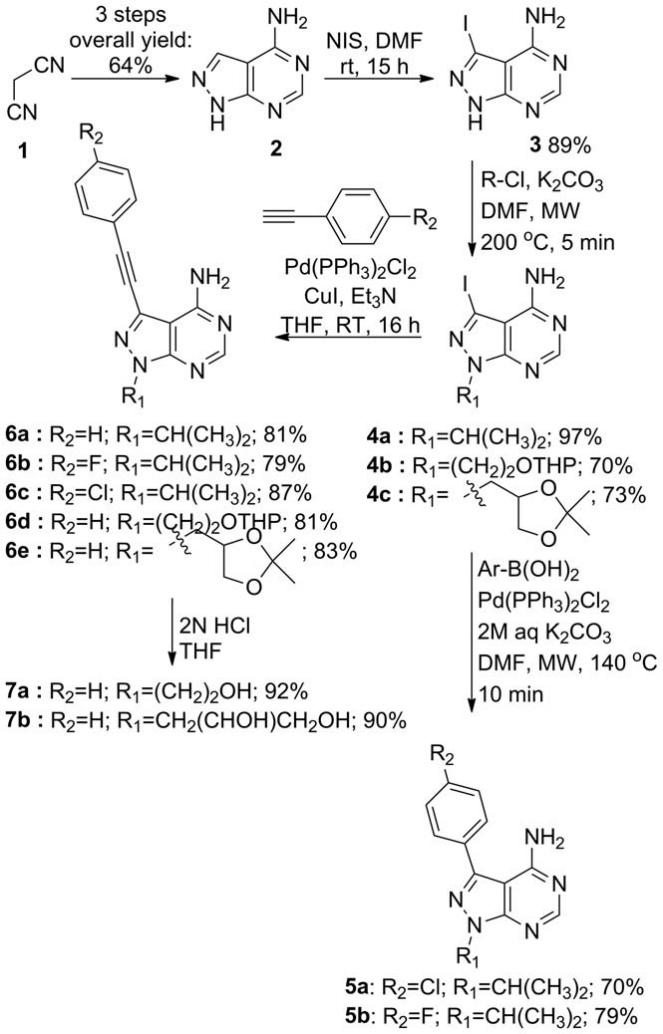
Synthesis of 1,3-disubstituted pyrazolopyrimidines.

Previous published synthetic approaches for making 1,3-disubstituted pyrazolopyrimidines involves at least five sequential reaction steps, but more importantly, the R_1_ substituent is introduced in the first step [17], [18]. Therefore, the generation of analogues with varying C3 substituents is inefficient. We devised a convergent route for making 1,3-disubstituted pyrazolopyrimidines. This route involves the synthesis of a common intermediate, 4-amino-3-iodo-1*H*-pyrazolo[3,4-*d*]pyrimidine (**3**) that allows rapid derivatization of the heterocyclic core scaffold in two steps (Figure 2).

The common intermediate, 4-amino-pyrazolopyrimidine (**2**), was synthesized from (**1**) by a 4-step synthesis, on a multigram scale in 64% overall yield without the use of any chromatography. The corresponding 4-amino-3-iodopyrazolopyrimidine (**3**) was synthesized using *N*-iodosuccinimide (Figure 2) [19].

Starting from compound **3**, a two-step derivatization process was developed. Initially, we found that alkylation of the N1 with 2-propanol using Mitsunobu conditions preceded only in moderate yield (58%). Treatment of **3** with 1.1 equiv of 2-chloropropan and 1.2 equiv of NaH in DMF for 3 h at 100°C gave a mixture of the N1 and N4 alkylated products. However, when the base was exchanged with K_2_CO_3_ and the reaction was carried out with microwave assisted heating at 200°C for 5 min, high region-selectivity was achieved and **4a** was obtained in nearly quantitative yield. Using the same reaction conditions in combination with commercial available 2-(2-Chloroethoxy)tetrahydro-2H-pyran or 4-Chloromethyl-2,2-dimethyl-1,3-dioxolane compounds **4b** and **4c** were obtained in 70% and 79% yield respectively.

Compound **4** was then reacted with 4-halogenphenyl boronic acid (1.3 eq) Pd(PPh_3_)_2_Cl_2_, (3 mol %), 2M aq. K_2_CO_3_ (10 eq) in THF at reflux for 16 h to generate compounds with the general structure **5** [17] or with 1-ethenyl-4-halobenzene, Pd(PPh_3_)_2_Cl_2_, (3 mol %), Et_3_N (2 eq) and CuI (6 mol %) in THF at reflux for 16 h to generate compounds with the general structure **6** [20]. In all cases the reactions proceeded with high turnover of the starting material and the target compounds were obtained in high yields (75–90%). Removal of the acid labile protection groups in **6d** and **6e** were carried out with 2N HCl in THF, resulting in compound **7a** and **7b** in 92% and 90% isolated yield respectively.

Using standard methods, we cloned, expressed and purified the glutathione-S-transferase (GST) fusion proteins of Hog1wt and Pbs2wt as well as the mutated kinases (Hog1as and Pbs2as). The analogue sensitive mutant allele of each kinase (*as* mutant) was created by the replacement of a conserved bulky residue with a glycine (T100G) in Hog1 or an alanine (M435A) in the active site of Pbs2.

**Figure 3:**
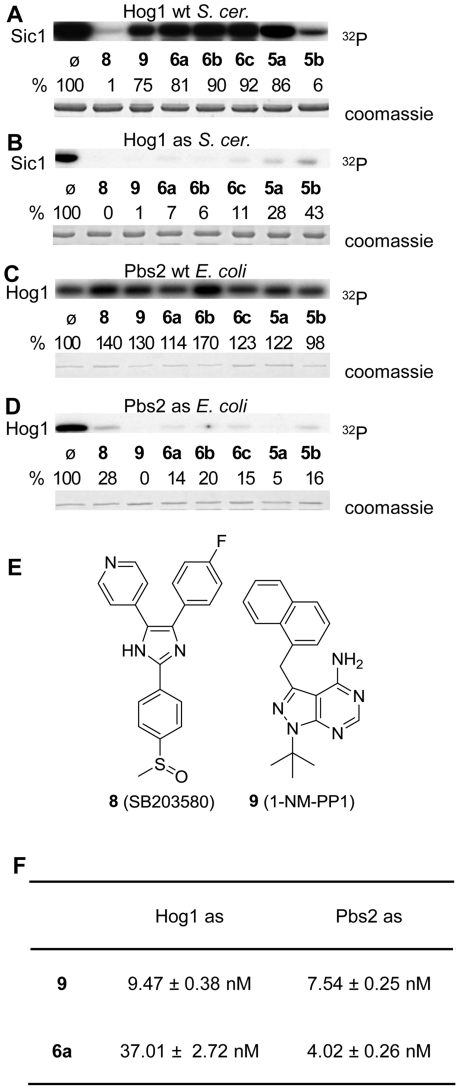
In vitro inhibition of Hog1 and Pbs2. *In vitro* inhibition of Hog1as (B) and Pbs2as (D) mutant variants by inhibitors, and effect in the wild type partners (A and C). The inhibitors used [final concentration 5 µM] were **6a**–**6c**, **5a**, **5b**, as well as SB203580 (**8**) (a known inhibitor of p38α, p38β, p38β2 and AKT/PKB) and 1NMPP1 (**9**), a known inhibitor of the as kinases. Recombinant, tagged proteins were purified either from *S. cerevisiae* (Hog1) or *E.coli* (Pbs2) and were assayed for the phosphorylation of Sic1 (substrate of Hog1) or Hog1 (as substrate of Pbs2). Phosphorylated proteins were resolved by SDS-PAGE and their phospho-state detected by autoradiography. IC_50_ values for *in vitro* inhibition of Hog1as and Pbs2as mutant variants by **6a** and **9** (F). Recombinant tagged proteins were purified either from *S. cerevisiae* (Hog1) or *E.coli* (Pbs2) and were assayed for phosphorylation of Sic1 (substrate of Hog1) or Hog1 (as substrate of Pbs2). The results are the means ± S.D. of at least three independent experiments.

Based on the analyses of their *in vitro* kinase activity, both Hog1as and Pbs2as were more active when the phosphodonor is the analogue Phenyl-Ethyl-ATP (PE-ATP) versus ATP, consistent with previous reports [5]. It is worth noting that, Hog1 expressed from *E. coli* was not inhibited by 1-NM-PP1 (**9**, Figure 3) [16], [21], [22], [23], while Hog1 produced from yeast was sensitive to this inhibitor (data not shown). This observation suggests the E. coli protein is inactive/only partially active and that full function of Hog1 depends on association with another yeast protein, such as a chaperone or that the conformation or postranslational modification of the Hog1 protein in *E. coli* is different from that produced in yeast.

We tested the 1,3-disubstituted pyrazolopyrimidines (**6a**–**6c**, **5a**, **5b**), as well as SB203580 (**8**, a known inhibitor of p38α, p38β and AKT/PKB. p38 is the mammalian homologue of Hog1) and 1-NM-PP1 (**9**, Figure 3). Hog1as was totally inhibited *in vitro* by **8** and **9**, and almost totally inhibited (>89%) by **6a**–**6c**, with the two last (**5a** and **5b**) the least efficient. Pbs2as was inhibited by all the compounds *in vitro*, but not as efficient as for Hog1 as. Protein levels were similar in all assays based on inspection of coomassie stained gels. As expected (as it is its homologue p38 in mammals), Hog1wt was inhibited by **8**, and poorly (range 8–25%) inhibited by the rest of compounds except for **5b** (94%). Finally, none of the inhibitors had an effect on Pbs2wt.

The *in vitro* IC_50_ values for compounds **9** and **6a** that target Hog1as and Pbs2as were determined (Figure 3F). Compound **9** was 4-times more efficient than **6a** when targeting Hog1as, while **6a** is less than two times more efficient when **9** when targeting Pbs2as. Thus, both inhibitors inhibited the kinases *in vitro* at a similar range of concentrations.

**Figure 4:**
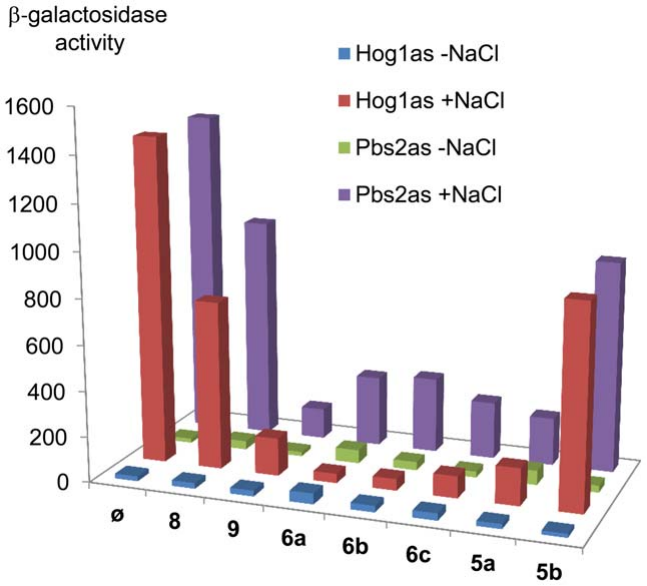
Transcription is inhibited by novel ATP analogues in response to stress. *hog1as* or *pbs2as* yeast cells were transformed with a *STL1::LacZ* reporter construct. Cells were grown to an OD_660_ of 0.2, incubated with the inhibitors for 8 hours at a concentration of 5 µM, and β-galactosidase activity was assayed before (−NaCl) or after (+NaCl) osmotic stress (0.4 M NaCl for 30 min). β-galactosidase activity is presented in nanomols per minute per milligram. Data represent the mean of at least three independent experiments.

As the mutated proteins were functional *in vitro*, yeast strains expressing the modified kinases were generated. Once we had checked that *hog1as* or *pbs2as* strains were osmoresistant (data not shown), we followed the transcriptional response to osmostress using the *STL1::LacZ* reporter (Figure 4). Cells were incubated with the different inhibitors for 8 hours at a fixed concentration of 5 µM, before an osmotic shock with 0.4 M NaCl. The results agreed with those observed in the *in vitro* assay, with the exception of **8**, that was less effective *in vivo*.

**Figure 5:**
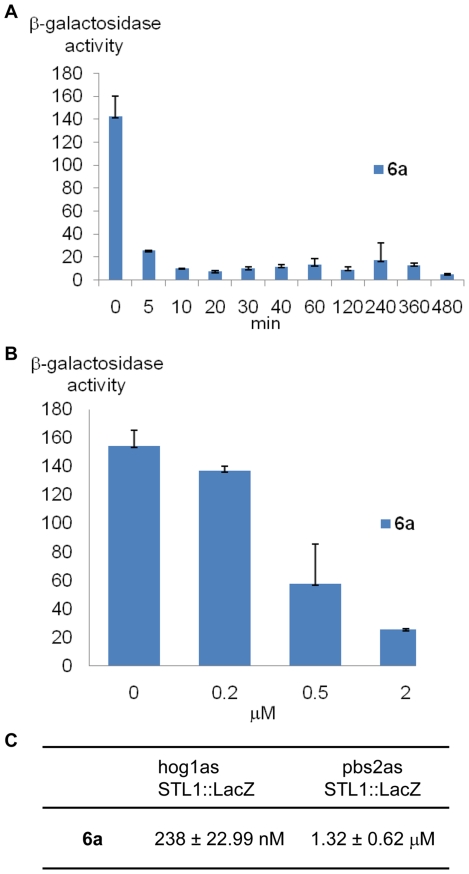
6a is efficient shortly after incubation. Time course at 2 µM concentration of (A) **6a** in the *hog1as* strain. *hog1as* cells transformed with the *STL1::LacZ* reporter were incubated with 2 µM of each inhibitor, subjected to osmotic stress (0.4 M NaCl), and cells were collected at different times. β-galactosidase activity is presented as fold induction of control versus NaCl-treated cells. (B) Dose response curve at 5 minutes of incubation with **6a**. *hog1as* cells were treated with different concentrations of **6a** for 5 minutes, subjected to osmotic shock and β-galactosidase activity was assayed as described above activity given in nanomols per minute per milligram). Efficiency of *in vivo* inhibition of *hog1as* and *pbs2as* strains by **6a** (C). *hog1as STL1::LacZ* or Pbs2as *STL1::LacZ* yeast cells were incubated with the inhibitors for 5 minutes, and β-galactosidase activity was assayed before (−NaCl) or after (+NaCl) osmostress (0.4 M NaCl for 30 min). Data±s.d. from at least three independent experiments are shown.

To further characterize **6a** we performed a time course at a concentration of 2 µM (end-point analysis), a concentration that does not manifest a fitness defect in a *HOG1* strain, but is sufficient to almost totally inhibit Hog1as (Figure 5 and data not shown). At 5 minutes of incubation with **6a** the β-galactosidase activity was inhibit by 86%, with an IC_50_ of 238 nM (Figure 5A and 5C). This rapid inhibition of gene expression by **6a** suggests that this compound could be a key, fast-acting tool to study some of the aspects of the mRNA biogenesis regulated specifically by the Hog1 MAPK as by signaling kinases in general.

**Figure 6:**
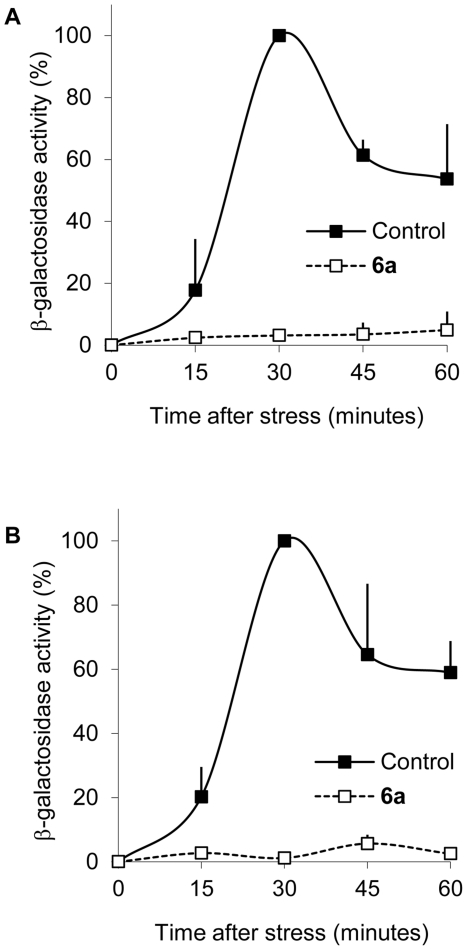
Inhibition over stress time course. *hog1as STL1::LacZ* (A) *and pbs2as STL1::LacZ* (B) cells were incubated with 5 µM of **6a** for 5 minutes and then, subjected to osmostress (0.4 M NaCl). Cells were collected at the indicated times. β-galactosidase activity is given as % of fold induction of control versus NaCl-treated cells. Data±s.d. from three independent experiments are shown.

To complement the end-point analyses of the inhibitors effect on transcription we also carried out a time course experiment for both *hog1as* and *pbs2as* (Figure 6). The addition of **6a** five minutes before stress completely blocked the expression of *STL1*, which in the absence of the inhibitor, increased up to 30 minutes in response to osmostress. Similar results were obtained by inhibition of Hog1as and Pbs2as.

**Figure 7:**
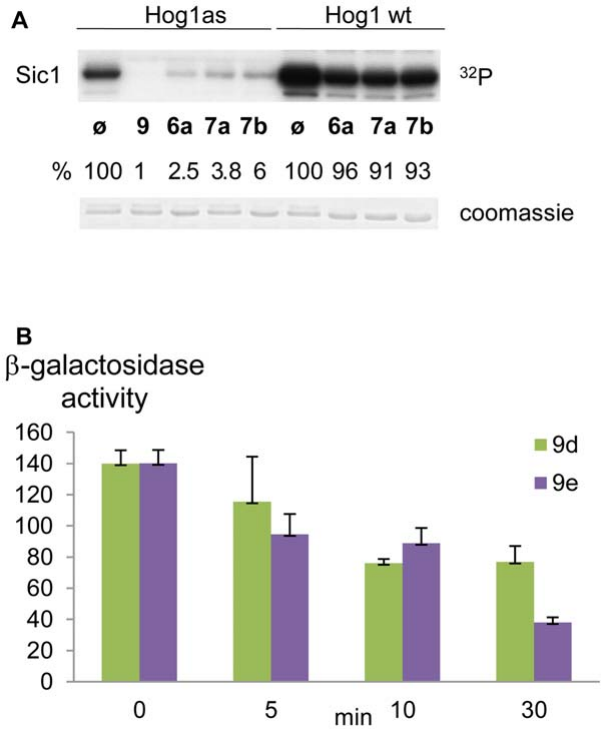
Hydroxylated derivates of 6a. (A) To increase the solubility of **6a** two derivatives **7a** and **7b** were synthesized. *In vitro*-activated Hog1 as was inhibited **7a** and **7b**. (B) *hog1as* cells transformed with the *STL1:LacZ* reporter were pre-incubated with 2 µM of **7a** and **7b** for the indicated times. Cells were subjected to osmotic stress (0.4 M NaCl) for 30 minutes and gene expression measured as before. β-galactosidase activity is given as fold induction of control versus NaCl-treated cells (activity given in nanomols per minute per milligram). Data±SD. from three independent experiments are shown.

To analyze whether the solubility of the molecule could affect the inhibition by **6a**, we tested two derivatives with one or two hydroxyl functions (**7a** and **7b**). We reasoned that increased solubility could result in better uptake and therefore more potent compounds. On the other hand, too polar compounds could be less bioactive if they are unable to cross the lipophilic cell membrane. However, we did not observe either a faster uptake or a faster inhibition with these two new derivates (Figure 7). Thus, although *in vitro* these compounds were able to inhibit Hog1as as efficiently as **6a** (Figure 7A), they did not improve the inhibition of the MAPK *in vivo* (Figure 7B).

The Stress-Activated Protein Kinase (SAPK) Hog1 elicits a program for cell adaptation that includes the control of gene expression and the modulation of cell-cycle progression. As recent studies have shown that monitoring SAPKs activity *in vivo* by reversable inhibition, we wanted to know if **6a**, is a suitable tool to study the transient cell cycle arrest mediated by Hog1 activation in response to stress.

**Figure 8:**
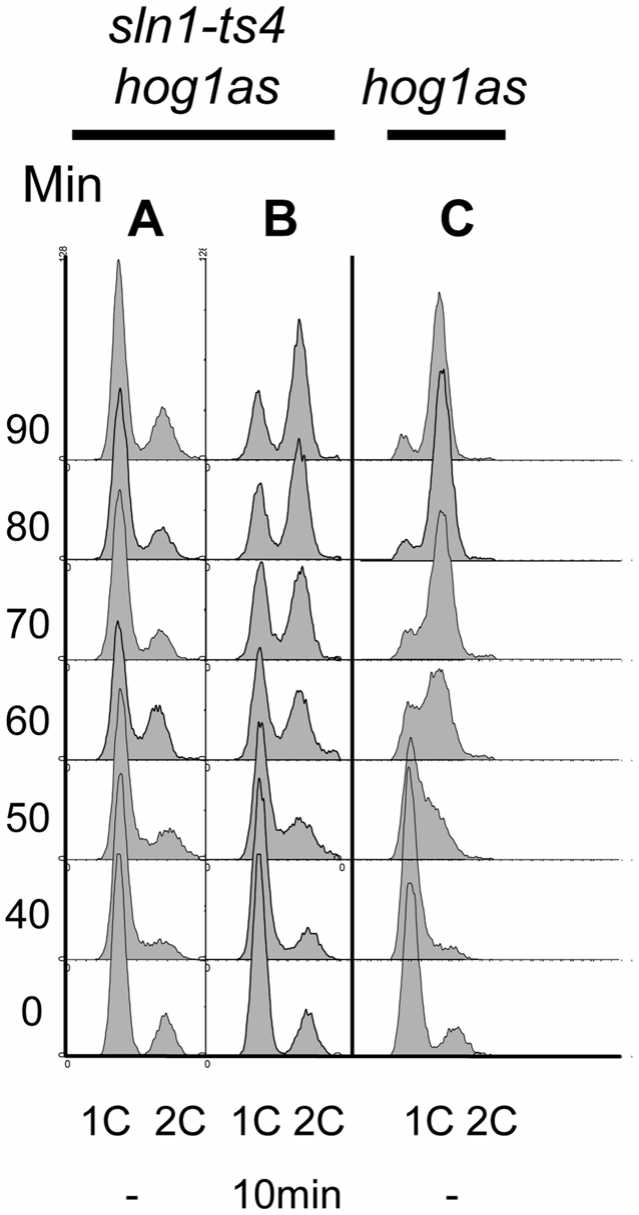
6a is a suitable tool to study the transient cell cycle arrest mediated by Hog1. The *sln1^ts4^ hog1as* or *hog1as* strains were synchronized with α-factor for 1 h, incubated with 5 µM of **6a** for 10 minutes (B), shifted to 37°C for 10 minutes and then released into YPD medium at 37°C plus the inhibitor (time 0). Total DNA content was assessed by flow cytometry and presented as cell counts (y-axis) versus 1C and 2C DNA content (x-axis).

Both high osmolarity and inactivation of Sln1 activity will result in activation of Hog1. It is known that cells manifest a transient cell cycle arrest in response to Sln1 inactivation, a phenotype that can be followed by flow cytometry [12], [24]. A temperature sensitive allele of *SLN1*, (*sln1^ts4^*) arrests at G_1_ phase following synchronization at G1/S with mating pheromone and release into the restrictive temperature (Figure 8, lane A). This arrest can be circumvented by mutations on the *HOG1* gene (in *hog1* cells) or if cells are pre-incubated with **6a** for as little as 10 min.

**Figure 9:**
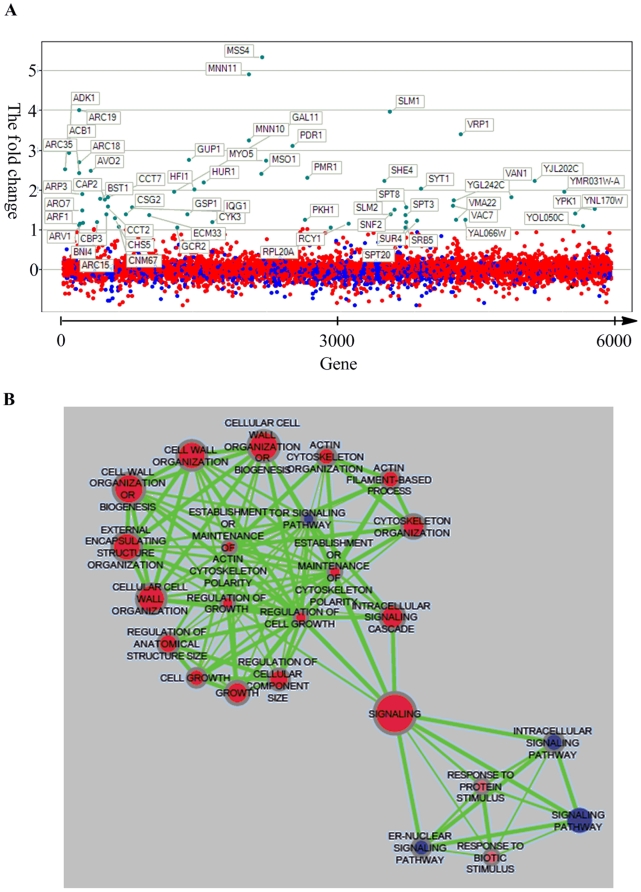
Chemical genetic profiling validation. (A) Visualization of genes sensitive to 500 µM **6a**. The fold change (log2(**6a**-treated/untreated)) in microarray signal intensity is plotted on the y-axis for ∼6000 genes (arranged alphabetically on the x-axis). (B) A network showing Kegg Pathways (Blue nodes) and related GO Terms (Red nodes) that are significantly enriched (FDR q-value <0.15) in the genes that are sensitive to the compound in the chemogenomic profile. Nodes are connected based on mutual overlap. Node size is proportional to the total number of genes in each set and edge thickness represents the number of overlapping genes between sets.

Our results demonstrate that **6a** is a powerful tool to study transient cell cycle arrest or gene expression mediated by Hog1 in response to stress. In addition, **6a** was recently used to demonstrate that dynamic signaling in the Hog1 pathway relies on high basal signal transduction [7]. However, the general applicability of this approach depends, in part, on the selectivity with **6a** inhibit the mutant protein kinases compared with the other wild-type protein kinases that are expressed endogenously in the same cells [25]. We therefore examined the specificity of **6a** by chemical genetic profiling of the yeast deletion mutant collection and scored for mutants with reduced growth in the presence of 500 µM **6a** and without osmotic stress (Figure 9). It should be noted that the concentration of **6a** used in this experiment was 100 times higher than what was required to get efficient inhibition of Hog1as (5 µM) in osmotic stressed cells and only off-target effects as well as secondary effects of these was expected to be identified. This analysis revealed that 60 strains that showed a significant depletion from the pool of 1200 essential heterozygotes and 4800 non-essential homozygous diploids (i.e. with a log2 ratio greater than 1) when cultured competitively in the presence of 500 µM **6a**. Notably, of the 60 gene deletion strains (see table S1, Supporting Information), 50 could be classified into five functional groups; kinases (6), other enzymes 14), cytoskeleton (17), transcription regulation (10), and cell wall (3), clearly demonstrating that several off-target effects takes place at this concentration of **6a**. A similar experiment has been reported using compound **9** (500 nM) targeting cdc28-as, showing excellent selectivity for the targeted kinase [26]. However, when the inhibitor concentration was increased to 5 µM several strains came up as sensitive, with several of these having a catalytic/nucleoside triphosphate binding role. Our results are consistent with these data. Together, these results demonstrate the importance of using the lowest possible dose and understanding the specific activity of each particular inhibitor.

To summarize, a potent, cell-permeable *as*-inhibitor of the yeast Hog1 MAP kinase has been developed and its utility has been demonstrated by studying various roles of the Hog1 kinase. The inhibitor can be regarded as a “sister-compound” of the commercial 1-NM-PP1. The inhibitor will be a very useful tool to study important model signal pathways in yeast.

## Materials and Methods

### Synthesis

^1^H and ^13^C NMR spectra were obtained from a JEOL JNM-EX 400 spectrometer. Column chromatography was performed on wet packed silica (0.040–0.063 mm) using flash chromatography. Microwave reactions were performed in a Biotage Initiator reactor with fixed hold time. Amino-3-iodo-1*H*-pyrazolo[3,4-*d*]pyrimidine was prepared following a literature procedure [19].

#### 1*H*-pyrazolo[3,4-*d*]pyrimidin-4-amine (2)

A solution of malononitrile (22.6 g, 0.344 mol), triethyl orthoformate (83 mL, 0.499 mol), and acetic anhydride (77 mL, 0.791 mol) was heated to 100°C for 5 h. After cooling to rt the solution was concentrated on the rotary evaporator. The solution was left to crystallize at rt overnight and the yellow solid was recrystallized from EtOH to give 36.0 g (98.5%) of 1,1-dicyano-2-ethoxylethene as yellow crystals. This material (36.0 g, 0.339 mol) was added carefully and in small portions to cold (0°C) hydrazine hydrate (99.5%, 26 g, 0.807 mol). The solution was heated to reflux for 1 h and then left to cool at rt whereupon the contents of the flask solidified. Water (25 mL) was added to the solid material and the mixture was left in the refrigerator overnight. The mixture was filtered and the solid was washed with 10 mL of cold water and suction dried for about 5 minutes. The product was dried in a vacuum desiccator over calcium chloride and was obtained in 75.5% (27.7 g). This material (27.6 g, 0.255 mol) was added to formamide (42 mL). The solution was vigorously boiled for 30 minutes (216°C oil bath). The creamy suspension was left to cool at rt, diluted with water (70 mL), and filtered. The light tan colored solid was washed with water, suction dried, and dried in a vacuum desiccator over calcium chloride to give the target compound in 86% yield (29.6 g) that gives an overall yield of 64% (calculated over three steps). NMR was in agreement with published data [27].

#### General procedure A; alkylation of N-1

Compound **3** (1 eq) and K_2_CO_3_ (2 eq) were suspended in 12 ml of dry DMF in a 20 ml microwave vessel. To this mixture, R_1_-Cl (1.1 eq) was added and the sealed tube was heated to 200°C for 5 min. (20 s of pre-stirring and fixed hold time: on). After cooling of the reaction mixture to room temperature, additional R_1_-Cl (0.5 eq) was added and the microwave vessel was heated again to 200°C for 5 min. After cooling to ambient temperature, the reaction mixture was diluted with DMF and filtered. The solvent was removed *in vacuo* at 80°C and the residue was co-distilled with toluene three times. The crude product was purified by flash column chromatography on silica gel.

#### 3-Iodo-1-isopropyl-1*H*-pyrazolo[3,4-*d*]pyrimidin-4-amine (4a)

Compound **3** (2.00 g, 7.66 mmol) was converted to the target compound using general procedure A. The crude product was purified by flash column chromatography on silica gel (MeOH∶CHCl_3_ = 1∶20) to give 97% (2.25 g) of **4a** as fine yellow needles. ^1^H NMR (400 MHz, CDCl_3_) δ 1.55 ppm (d, 6H), 5.09 (m, 1H), 6.17 (bs, 2H), 8.32 (s, 1H). ^13^C NMR (100 MHz, CDCl_3_) δ 22.27 ppm, 49.89, 85.62, 104.25, 153.17, 155.88, 157.63. Anal. Calcd for C_8_H_10_IN_5_ (303.00): C, 31.70; H, 3.33; N, 23.11. Found: C, 31.84; H, 3.40; N, 23.19.

#### 3-Iodo-1-(2-(tetrahydro-2H-pyran-2-yloxy)ethyl)-1*H*-pyrafzolo[3,4-*d*]pyrimidin-4-amine (4b)

Compound **3** (2.00 g, 7.66 mmol) was converted to the target compound using general procedure A. The crude product was purified by flash column chromatography on silica gel (MeOH∶CHCl_3_ = 1∶20) to give 70% (2.09 g) of **4b** as a yellow powder. ^1^H NMR (400 MHz, CDCl_3_) δ 1.27–1.64 ppm (m, 6H), 3.30–3.39 (m, 1H), 3.48–3.58 (m, 1H), 3.77–3.86 (m, 1H), 3.99–4.09 (m, 1H), 4.42–4.59 (m, 3H), 8.22 (s, 1H). ^13^C NMR (100 MHz, CDCl_3_) δ 18.93 ppm, 25.30, 30.21, 47.26, 61.71, 64.85, 86.59, 98.12, 103.77, 154.14, 155.79, 157.89. Anal. Calcd for C_12_H_16_IN_5_O_2_ (389.03): C, 37.03; H, 4.14; N, 17.99. Found: C, 37.04; H, 4.15; N, 18.04.

#### 1-((2,2-Dimethyl-1,3-dioxolan-4-yl)methyl)-3-iodo-1*H*-pyrazolo[3,4-*d*]pyrimidin-4-amine (4c)

Compound **3** (2.00 g, 7.66 mmol) was converted to the target compound using general procedure A. The crude product was purified by flash column chromatography on silica gel (MeOH∶CHCl_3_ = 1∶20) to give 73% (2.09 g) of **4c** as fine yellow needles. ^1^H NMR (400 MHz, CDCl_3_) δ 1.33 ppm (d, 6H), 1.40 (s, 3H), 3.92–3.96 (m, 1H), 4.04–4.08 (m, 1H), 4.56–4.65 (m, 2H), 6.24 (bs, 2H), 8.34 (s, 1H). ^13^C NMR (100 MHz, CDCl_3_) δ 25.52 ppm, 27.00, 50.12, 67.34, 74.28, 86.91, 104.17, 110.17, 154.59, 156.45, 157.69. Anal. Calcd for C_11_H_14_IN_5_O_2_ (375.02): C, 35.22; H, 3.76; N, 18.67. Found: C, 35.24; H, 3.79; N, 18.65.

#### General procedure B; Suzuki couplings

Compound **4** (1 eq), Ar-B(OH)_2_ (1.3 eq) and Pd(PPh_3_)_2_Cl_2_ (3 mol %) were suspended in DMF (2 ml) and 2 M aq K_2_CO_3_ added. The reaction vial was sealed and heated in a microwave reactor to 140°C for 10 min. After cooling to ambient temperature, the reaction mixture was diluted with DMF and filtered. The solvent was removed *in vacuo* and the residue was co-distilled with toluene three times. The crude product was purified by flash column chromatography on silica gel.

#### 3-(4-Chlorophenyl)-1-isopropyl-1*H*-pyrazolo[3,4-*d*]pyrimidin-4-amine (5a)

Compound **4a** (2.00 g, 6.60 mmol) was converted to the target compound using general procedure B. The crude product was purified by flash column chromatography on silica gel (MeOH∶CHCl_3_ = 1∶35) to give 70% (1.33 g) of **5a** as fine yellow needles.^1^H NMR (400 MHz, CDCl_3_) δ 1.59 ppm (d, 6H), 5.18 (m, 1H,), 6.17 (bs, 2H), 7.48–7.54 (m, 2H), 7.63–7.68 (m, 2H), 8.35 (s, 1H). ^13^C NMR (100 MHz, CDCl_3_) δ 22.19 ppm, 46.16, 98.71, 129.70, 130.03, 135.28, 142.78, 173.77, 155.62, 158.10. Anal. Calcd for C_14_H_14_ClN_5_ (287.75): C, 58.44; H, 4.90; N, 24.34. Found: C, 58.46; H, 4.91; N, 24.36.

#### 3-(4-Fluorophenyl)-1-isopropyl-1*H*-pyrazolo[3,4-*d*]pyrimidin-4-amine (5b)

Compound **4a** (2.00 g, 6.60 mmol) was converted to the target compound using general procedure A. The crude product was purified by flash column chromatography on silica gel (MeOH∶CHCl_3_ = 1∶35) to give 79% (1.41 g) of **5b** as fine yellow needles. ^1^H NMR (400 MHz, CDCl_3_) δ 1.58 ppm (d, 6H), 5.16 (m, 1H,), 6.19 (bs, 2H), 7.18–7.24 (m, 2H), 7.66–7.71 (m, 2H), 8.32 (s, 1H). ^13^C NMR (100 MHz, CDCl_3_) δ 22.06 ppm, 48.96, 98.58, 116.42, 129.77, 130.46, 142.89, 153.52, 155.41, 158.16, 163.24. Anal. Calcd for C_14_H_14_FN_5_ (271.30): C, 61.98; H, 5.20; N, 25.81. Found: C, 61.99; H, 5.22; N, 25.84.

#### General procedure C; Sonogashira couplings

Compound **4a** (1 eq), Ar-CCH (1.3 eq), Pd(PPh_3_)_2_Cl_2_ (3 mol %) Et_3_N (2 eq) and CuI (6 mol %) in THF was refluxed for 16 h. After cooling to ambient temperature, the reaction mixture was filtered. The solvent was removed *in vacuo* and the residue was co-distilled with toluene three times. The crude product was purified by flash column chromatography on silica gel.

#### 1-Isopropyl-3-(phenylethynyl)-1*H*-pyrazolo[3,4-*d*]pyrimidin-4-amine (6a)

Compound **4a** (0.20 g, 0.66 mmol) was converted to the target compound using general procedure C. The crude product was purified by flash column chromatography on silica gel (MeOH∶CHCl_3_ = 1∶30) to give 81% yield (148 mg) of **6a** as fine white powder. ^1^H NMR (400 MHz, CDCl_3_) δ 1.55 ppm (d, 6H), 5.09 (m, 1H), 6.17 (bs, 2H), 8.32 (s, 1H). ^13^C NMR (100 MHz, CDCl_3_) δ 22.27 ppm, 49.89, 85.62, 104.25, 153.17, 155.88, 157.63. Anal. Calcd for C_16_H_15_N_5_ (277.13): C, 69.29; H, 5.45; N, 25.25. Found: C, 69.35; H, 5.46; N, 25.21.

#### 3-((4-fluorophenyl)ethynyl)-1-isopropyl-1*H*-pyrazolo[3,4-*d*]pyrimidin-4-amine (6b)

Compound **4a** (0.20 g, 0.66mmol) was converted to the target compound using general procedure C. The crude product was purified by flash column chromatography on silica gel (MeOH∶CHCl_3_ = 1∶30) to give 79% yield (154 mg) of **6b** as fine white powder. ^1^H NMR (400 MHz, CDCl_3_) δ 1.58 ppm (d, 6H), 5.16 (m, 1H), 6.22 (bs, 2H), 7.07–7.11 (m, 2H), 7.56–7.60 (m, 2H), 8.36 (s, 1H). ^13^C NMR (100 MHz, CDCl_3_) δ 22.20 ppm, 49.67, 80.97, 92.91, 102.07, 116.23, 117.92, 125.92, 134.04, 152.71, 156.37, 158.02, 163.32. Anal. Calcd for C_16_H_14_FN_5_ (295.30): C, 65.07; H, 4.78; N, 23.71. Found: C, 65.10; H, 4.73; N, 23.77.

#### 3-((4-chlorophenyl)ethynyl)-1-isopropyl-1*H*-pyrazolo[3,4-*d*]pyrimidin-4-amine (6c)

Compound **4a** (0.20 g, 0.66mmol) was converted to the target compound using general procedure C. The crude product was purified by flash column chromatography on silica gel (MeOH∶CHCl_3_ = 1∶30) to give 87% yield (179 mg) of **6c** as a white powder. ^1^H NMR (400 MHz, CDCl_3_) δ 1.46 ppm (d, 6H), 5.04 (m, 1H), 7.54 (d, 2H), 7.78 (d, 2H), 8.25 (s, 1H). ^13^C NMR (100 MHz, CDCl_3_) δ 21.71 ppm, 48.76, 82.22, 91.69, 120.34, 124.77, 128.77, 133.61, 134.11, 152.28, 156.21, 157.71. Anal. Calcd for C_16_H_14_ClN_5_ (311.09): C, 61.64; H, 4.53; N, 22.46. Found: C, 22.46; H, 4.53; N, 22.42.

#### 3-(Phenylethynyl)-1-(2-(tetrahydro-2H-pyran-2-yloxy)ethyl)-1*H*-pyrazolo[3,4-*d*]pyrimidin-4-amine (6d)

Compound **4b** (0.20 g, 0.66 mmol) was converted to the target compound using general procedure C. The crude product was purified by flash column chromatography on silica gel (MeOH∶CHCl_3_ = 1∶30) to give 81% yield (194 mg) of **6d** as a slightly yellow powder. ^1^H NMR (400 MHz, CDCl_3_) δ 1.38–1.75 ppm (d, 6H), 3.41–3.52 (m, 1H), 3.62–3.70 (m, 1H) 3.91–3.99 (m, 1H), 4.14–4.22 (m, 1H), 4.56–4.71 (m, 3H), 5.93 (bs, 2H), 7.38–7.47 (m, 3H), 7.58–7.63 (m, 2H), 8.40 (s, 1H). ^13^C NMR (100 MHz, CDCl_3_) δ 19.10 ppm, 25.48, 30.42. 47.47, 61.94, 65.00, 80.93, 94.26, 98.40, 101.85, 121.67, 126, 74, 128.78, 129.64, 131.94, 154.04, 156.60, 158.10. Anal. Calcd for C_20_H_21_N_5_O_2_ (363.17): C, 66.10; H, 5.82; N, 19.27. Found: C, 66.12; H, 5.83; N, 19.30.

#### 1-((2,2-dimethyl-1,3-dioxolan-4-yl)methyl)-3-(phenylethynyl)-1*H*-pyrazolo[3,4-*d*]pyrimidin-4-amine (6e)

Compound **4c** (0.20 g, 0.66 mmol) was converted to the target compound using general procedure C. The crude product was purified by flash column chromatography on silica gel (MeOH∶CHCl_3_ = 1∶20) to give 83% yield (191 mg) of **6e** as a white powder. ^1^H NMR (400 MHz, CDCl_3_) δ 1.34 ppm (d, 3H), 1.40 (s, 3H), 3.94–4.00 (m, 1H), 4.06–4.11 (m, 1H) 4.43–4.52 (m, 1H), 4.58–4.69 (m, 2H), 6.20 (bs, 2H), 7.37–7.46 (m, 3H), 7.57–7.62 (m, 2H), 8.39 (s, 1H). ^13^C NMR (100 MHz, CDCl_3_) δ 25.52 ppm, 27.01, 50,09, 67.42, 74.25, 80.80, 94.56, 101.90, 110.18, 121.60, 127.14, 128.85, 129.78, 132.01, 154.12, 156.93, 157.99. Anal. Calcd for C_19_H_19_N_5_O_2_ (349.15): C, 65.32; H, 5.48; N, 20.04. Found: C, 65.35; H, 5.50; N, 20.09.

#### General procedure D; deprotection of acid labile groups

The starting material (1 eq) was dissolved in 9 ml THF and 6 ml of 2N HCl was added. The mixture was stirred at 60°C for 2 h. The solvent was evaporated and the residue co-evaporated with toluene three times. The product was purified by crystallization from MeOH/Et_2_O.

#### 2-(4-amino-3-(phenylethynyl)-1*H*-pyrazolo[3,4-*d*]pyrimidin-1-yl)etanol (7a)

Compound **6d** (0.15 g, 0.48 mmol) was converted to the target compound using general procedure C and was obtained in 92% yield (120 mg) of **7a** as a white powder. ^1^H NMR (400 MHz, DMSO) δ 3.84 ppm (t, 2H), 4.22 (t, 2H), 7.45–7.54 (m, 3H), 7.73–7.79 (m, 2H), 8.49 (s, 1H). ^13^C NMR (100 MHz, DMSO) δ 50.18 ppm, 59.01, 79.72, 94.50, 99.93, 120.98, 127.37, 128.68, 129.79, 132.06, 150.22, 151.82, 153.51. Anal. Calcd for C_15_H_14_ClN_5_O (315.09): C, 57.05; H, 4.47; N, 22.18. Found: C, 57.10; H, 4.51; N, 22.24.

#### 3-(4-amino-3-(phenylethynyl)-1*H*-pyrazolo[3,4-*d*]pyrimidin-1-yl)propane-1,2-diol (7b)

Compound **6d** (0.15 g, 0.43 mmol) was converted to the target compound using general procedure C and was obtained in 90% yield (133 mg) of **7b** as a white powder. ^1^H NMR (400 MHz, DMSO) δ 3.37–3.50 ppm (m, 2H), 3.96–4.05 (m, 1H), 4.32–4.43 (m, 2H), 7.45–7.54 (m, 3H), 7.74–7.80 (m, 2H), 8.39 (s, 1H). ^13^C NMR (100 MHz, DMSO) δ 51.17 ppm, 63.61, 69.95, 79.57, 94.67, 99.82, 120.94, 127.55, 128.67, 129.82, 132.09, 149.35, 151.66, 152.99. Anal. Calcd for C_16_H_16_ClN_5_O_2_ (345.10): C, 55.58; H, 4.66; N, 20.25. Found: C, 55.65; H, 4.70; N, 20.28.

### Biology

#### Yeast strains and plasmids

Strains used: W303 (MATa *ade2-1 ura3-1 leu2-3,112 trp1-1 his3-11 can1-100 GAL SUC2*) and its derivatives, W303 *hog1-as* strain (MATa *ade2-1 ura3-1 leu2-3,112 trp1-1 his3-11 can1-100 GAL SUC2 hog1-as*) and W303 *pbs2-as* strain (MATa *ade2-1 ura3-1 leu2-3,112 trp1-1 his3-11 can1-100 GAL SUC2 pbs2::URA pbs2-as:TRP*). The pRS426TEG1 (p_TEF1_-*GST*, *URA3* ^+^, 2 µm) vector was a gift from M. Takekawa. The pRS426TEG1-*hog1as* was created by cloning the *hog1* mutant ORF plus 500 bp upstream and downstream, into pRS426TEG1 empty plasmid. To build the as mutant the *hog1* orf was mutated by PCR in the amino acid 100 (T-ACG-→G-GGG- ). The pRS304-Pbs2as vector was created by cloning the pbs2 mutant ORF plus 500 bp upstream and downstream, into pRS304 empty plasmid. To build the as mutant the *pbs*2 orf was mutated by PCR in the amino acid 435 (M-ATG-→A-GCG-). *ssk2ΔN*, *pbs2^EE^*, HOG1, PBS2, *pbs2-as* and SIC1 ORFs were cloned into pGEX-4T1 (Pharmacia) to obtain GST fusion proteins. The *STL1*::*LacZ* reporter construct PEN05 was generated by cloning the *STL1* promoter (base pairs −824 to +4) by PCR into YIp358R (*CEN URA3*).

#### Expression and purification of epitope-tagged proteins

GST fusion proteins were expressed in *E.coli* DH5α, and purified using glutathione-Sepharose beads (Pharmacia) in buffer B as described [28]. The beads were washed extensively with buffer B and finally proteins were eluted with kinase buffer plus glutathione (50 mM Tris-HCl pH 8.0, 2 mM DTT, 10 mM glutathione). GST-Hog1as was expressed in *S. cerevisiae* and purified using glutathione-Sepharose beads (Pharmacia) in buffer B, as described above.

#### In vitro phosphorylation experiments

One microgram of recombinant GST-Hog1 or GST-Pbs2 was activated by phosphorylation using 0.5 µg of either GST-Pbs2^EE^ or GST-Ssk2ΔN in the presence of kinase buffer and ATP as described [28]. After 20 min at 30°C, substrate was added (GST-Sic1 for Hog1 or GST-Hog1 for Pbs2) to the previous mixture together with [γ-^32^P]-ATP (for wt kinases) or [γ-^32^P]-phenethyl-ATP (for as kinases) (5 µCi). The mixture was then incubated for 10 min at 30°C, and the reactions were terminated by the addition of 5× SDS loading buffer. Labeled proteins were resolved by SDS-PAGE and detected by autoradiography. Inhibitors were added to the first mixture at the indicated concentration. The assessment of the IC_50_ was performed by the quantification of phosphorylated substrates (Sic1 or Hog1) by using Quantity One software (BioRad).

#### β-galactosidase assays

Exponentially growing cells (OD_660_ = 0.8) were incubated with the specified inhibitor previous to be subjected to osmotic stress (0.4 M NaCl for 30 min). Cells were permeabilized by ethanol-toluene treatment, and β-galactosidase was measured as described [29]. Results are presented as mean values obtained from two independent transformants measured at least in triplicate.

#### Cytometry analyses

For flow cytometry analyses, cells were fixed in ethanol, treated with RNAse A, stained with propidium iodide and analysed in a FACScan flow cytometer (Becton Dickinson) in the FL3 channel. A total of 10000 cells were analysed for each time point.

#### Chemical Genetic Profiling

Yeast profiling was performed exactly as described by Ericson *et al* [30]. Gene set enrichment was carried out on the non-essential yeast genes in the chemogenomic profile using GSEA [31] and the results were visualized as a network using the enrichment map plugin [32] for cytoscape [33].

### Modeling

#### MolIDE/ICM modeling

The initial model of Hog1 was created using the standard procedure built into the program ICM, based on the structure of human p38 (pdb code 1p38) [34]. The model was refined by regularization, which imposes ideal geometry onto the model, followed by geometry optimization by Monte Carlo simulated annealing in torsion space. (ICM manual, Molsoft, CA) Ligands were manually docked into the site and geometry optimized by minimization and Monte Carlo conformation sampling. In the early phase of optimization, ligand atoms were tethered to their original positions using harmonic potentials that were gradually decreased in strength.

Eight additional wild-type and mutant Hog1 models were created based on four structure templates (pdb codes 1cm8, 1m7q, 1p38 and 3erk) to provide a better understanding of the orthogonal ligands' affinities. Template selection was carried out using the MolIDE program [35], which is an interface to programs that performs psi-blast searches against the nr database and stores the generated alignment profiles, searches the pdb sequences using the profiles, constructs a backbone model for aligned residues, adds and geometry optimizes side-chains and builds loops. Pairwise sequence alignments were manually edited before model building.

Kinase models built by MolIDE were regularized by ICM before docking and quality assessment. The local quality of protein models was checked using ICM's calcEnergyStrain, which identifies bad regions by reporting the relative energy of each residue.

Docking of a small library of the designed and known kinase inhibitors was performed using the standard protocol in ICM (data not shown), and the representative mutant and wild-type Hog1 models were manually chosen based on the accuracy of ligand docking poses.

## Supporting Information

Table S1 **Gene deletion strains significantly sensitive to 500 uM 6a.**
(PDF)
