# Supplementary material for: Design, Synthesis and Characterization of a Highly Effective Inhibitor for Analog-Sensitive (as) Kinases
Source: PLoS One. 2011 Jun 17;6(6):e20789. doi: 10.1371/journal.pone.0020789 (PMC3117834; doi:10.1371/journal.pone.0020789)
Supplement: Table S1 — Gene deletion strains significantly sensitive to 500 uM 6a. (PDF) [file pone.0020789.s001.pdf]

kinase
other enzyme
cytoskeleton
transcriptional regulation
cell wall

probeid	6a	gene	essential	feature_type	GO_process	GO_function	GO_component
YDR208W::chr4_5	5,339	MSS4	yes	ORF::Verified	sporulation (sensu Fungi)*	1-phosphatidylinositol-4-phosphate 5-kinase activity	plasma membrane
YJL183W::chr10_1	4,898	MNN11	no	ORF::Verified	protein amino acid glycosylation	alpha-1,6-mannosyltransferase activity	alpha-1,6-mannosyltransferase complex
YKL013C::chr11_1	4,01	ARC19	yes	ORF::Verified	mitochondrion inheritance*	structural molecule activity	Arp2/3 protein complex
YIL105C::chr9_2	3,976	SLM1	no	ORF::Verified	actin cytoskeleton organization and biogenesis*	phosphoinositide binding	cytoplasm*
YLR337C::chr12_5	3,394	VRP1	no	ORF::Verified	endocytosis*	actin binding	actin cortical patch
YDR245W::chr4_6	3,252	MNN10	no	ORF::Verified	actin filament organization*	alpha-1,6-mannosyltransferase activity	alpha-1,6-mannosyltransferase complex
YGL013C::chr7_1	3,101	PDR1	no	ORF::Verified	regulation of transcription from RNA polymerase II promoter*	DNA binding*	nucleus
YDR226W::chr4_5	2,925	ADK1	no	ORF::Verified	nucleotide metabolism*	adenylate kinase activity	cytoplasm*
YGL084C::chr7_1	2,752	GUP1	no	ORF::Verified	telomere maintenance*	O-acyltransferase activity	endoplasmic reticulum*
YMR109W::chr00_9	2,734	MYO5	no	ORF::Verified	cell wall organization and biogenesis*	microfilament motor activity	actin cortical patch
YLR370C::chr00_11	2,69	ARC18	no	ORF::Verified	actin filament organization*	structural constituent of cytoskeleton	Arp2/3 protein complex
YGR037C::chr7_4	2,516	ACB1	no	ORF::Verified	fatty acid metabolism*	long-chain fatty acid transporter activity*	cytoplasm*
YMR068W::chr00_6	2,476	AVO2	no	ORF::Verified	regulation of cell growth*	molecular function unknown	cytoplasm*

YNR035C::chr14_4	2,425	ARC35	yes	ORF::Verified	actin cytoskeleton organization and biogenesis*	structural molecule activity	cytosol*
YNR049C::chr14_4	2,404	MSO1	no	ORF::Verified	sporulation (sensu Fungi)*	molecular function unknown	microsome
YGL167C::chr7_2	2,303	PMR1	no	ORF::Verified	secretory pathway*	calcium-transporting ATPase activity*	Golgi apparatus
YJL202C::chr10_1	2,223	YJL202C	yes	ORF::Dubious			
YOR035C::chr15_1	2,223	SHE4	no	ORF::Verified	actin cytoskeleton organization and biogenesis*	myosin binding	cytoplasm
YGL168W::chr7_2	2,183	HUR1	no	ORF::Uncharacterized	telomere maintenance*	molecular function unknown	nucleus
YPR095C::chr16_4	2,032	SYT1	no	ORF::Verified	vesicle-mediated transport*	ARF guanyl-nucleotide exchange factor activity	mitochondrion
YPL254W::chr16_1	2,022	HFI1	no	ORF::Verified	telomere maintenance*	transcription coactivator activity*	SAGA complex*
YOL051W::chr15_5	1,966	GAL11	no	ORF::Verified	telomere maintenance*	RNA polymerase II transcription mediator activity	mediator complex
YMR031W-A::chr13_2	1,95	YMR031W-A	no	ORF::Dubious			
YJR065C::chr00_13	1,908	ARP3	yes	ORF::Verified	actin filament organization*	structural constituent of cytoskeleton*	Arp2/3 protein complex
YJL111W::chr10_2	1,816	CCT7	yes	ORF::Verified	protein folding*	unfolded protein binding	cytoplasm*
YGL242C::chr7_3	1,814	YGL242C	no	ORF::Uncharacterized	biological process unknown	molecular function unknown	cellular component unknown
YFL025C::chr6_1	1,773	BST1	no	ORF::Verified	ER to Golgi vesicle-mediated transport*	carboxylic ester hydrolase activity	endoplasmic reticulum*
YIL034C::chr9_1	1,765	CAP2	no	ORF::Verified	filamentous growth*	actin filament binding	actin cortical patch*
YML115C::chr00_15	1,741	VAN1	no	ORF::Verified	protein amino acid N-linked glycosylation	mannosyltransferase activity	membrane*
YNL054W::chr14_3	1,6	VAC7	no	ORF::Verified	vacuole inheritance*	enzyme regulator activity	cytoplasm*
YIL142W::chr9_2	1,577	CCT2	yes	ORF::Verified	protein folding*	unfolded protein binding	cytoplasm*

YLR055C::chr12_2	1,575	SPT8	no	ORF::Verified	positive regulation of transcription from RNA polymerase II promoter*	transcription cofactor activity*	nucleus*
YDL117W::chr4_2	1,559	CYK3	no	ORF::Verified	cytokinesis	molecular function unknown	cytoplasm*
YKL126W::chr11_2	1,531	YPK1	no	ORF::Verified	protein amino acid phosphorylation*	protein serine/threonine kinase activity	cytosol*
YOR290C::chr15_4	1,516	SNF2	no	ORF::Verified	chromatin remodeling*	general RNA polymerase II transcription factor activity*	SWI/SNF complex*
YPR060C::chr16_4	1,496	ARO7	no	ORF::Verified	aromatic amino acid family biosynthesis	chorismate mutase activity	cytoplasm*
YNL170W::chr14_2	1,417	YNL170W	no	ORF::Dubious			
YLR293C::chr12_4	1,397	GSP1	no	ORF::Verified	rRNA processing*	GTPase activity	cytoplasm*
YBR036C::chr2_2	1,396	CSG2	no	ORF::Verified	calcium ion homeostasis*	enzyme regulator activity	integral to endoplasmic reticulum membrane
YNL047C::chr00_18	1,391	SLM2	no	ORF::Verified	actin cytoskeleton organization and biogenesis*	phosphoinositide binding	plasma membrane*
YPL215W::chr16_1	1,382	CBP3	no	ORF::Verified	protein complex assembly	molecular function unknown	mitochondrial envelope
YDR392W::chr4_7	1,369	SPT3	no	ORF::Verified	sporulation (sensu Fungi)*	transcription cofactor activity	SAGA complex*
YBR078W::chr2_2	1,362	ECM33	no	ORF::Verified	cell wall organization and biogenesis	molecular function unknown	mitochondrion*
YLR330W::chr12_5	1,298	CHS5	no	ORF::Verified	spore wall assembly (sensu Fungi)*	molecular function unknown	cytoplasm
YAL066W::chr1_1	1,252	YAL066W	no	ORF::Dubious			
YHR060W::chr8_2	1,25	VMA22	no	ORF::Verified	protein complex assembly*	unfolded protein binding	endoplasmic reticulum membrane
YDR490C::chr4_8	1,247	PKH1	no	ORF::Verified	protein amino acid phosphorylation*	protein kinase activity	cytosol
YLR372W::chr12_5	1,227	SUR4	no	ORF::Verified	telomere maintenance*	fatty acid elongase activity	endoplasmic reticulum*
YGR104C::chr	1,203	SRB5	no	ORF::Verified	telomere maintenance*	RNA polymerase II transcription	mediator complex

7_4						mediator activity	
YPL242C::chr 16_1	1,197	IQG1	yes	ORF::Verified	actin filament organization*	cytoskeletal protein binding	contractile ring (sensu Saccharomyces)
YNL233W::ch r14_2	1,196	BNI4	no	ORF::Verified	chitin biosynthesis*	protein binding	bud neck*
YLR242C::chr 12_4	1,169	ARV1	no	ORF::Verified	telomere maintenance*	molecular function unknown	endoplasmic reticulum*
YDL192W::ch r4_3	1,16	ARF1	no	ORF::Verified	telomere maintenance*	GTPase activity	cytosol*
YMR242C::ch r13_5	1,158	RPL20A	no	ORF::Verified	translation	structural constituent of ribosome	cytosolic large ribosomal subunit (sensu Eukaryota)
YIL062C::chr9 _1	1,123	ARC15	yes	ORF::Verified	mitochondrion inheritance*	structural molecule activity*	mitochondrial envelope*
YOL050C::chr 15_5	1,103	YOL050C	no	ORF::Dubious			
YNL225C::chr 14_2	1,086	CNM67	no	ORF::Verified	microtubule nucleation*	structural constituent of cytoskeleton	spindle pole body*
YOL148C::chr 00_18	1,06	SPT20	no	ORF::Verified	histone acetylation*	transcription cofactor activity	SAGA complex*
YJL204C::chr 10_1	1,053	RCY1	no	ORF::Verified	endocytosis	protein binding	Golgi apparatus*
YNL199C::chr 14_2	1,05	GCR2	no	ORF::Verified	positive regulation of transcription from RNA polymerase II promoter*	transcriptional activator activity	nucleus
YHR207C::chr 8_3	1,029	SET5	no	ORF::Verified	biological process unknown	molecular function unknown	cytoplasm*
YPL031C::chr 16_3	1,029	PHO85	no	ORF::Verified	telomere maintenance*	cyclin-dependent protein kinase activity	nucleus
YAL023C::chr 1_1	1,019	PMT2	no	ORF::Verified	protein amino acid O-linked glycosylation	dolichyl-phosphate-mannose-protein mannosyltransferase activity	endoplasmic reticulum
YPL161C::chr 16_2	1,012	BEM4	no	ORF::Verified	telomere maintenance*	molecular function unknown	cytoplasm*
YLR087C::chr 12_2	1,004	CSF1	no	ORF::Verified	fermentation	molecular function unknown	mitochondrion
